# BALF cellular composition in pediatric LRTI: from pathogen-specific patterns to clinical correlates

**DOI:** 10.3389/fcimb.2026.1946336

**Published:** 2026-09-29

**Authors:** Fen Liu, Jiapeng Hu, Jinling Wang, XiangTeng Liu, Qi Cheng, Lishen Shan, Jiujun Li, Ning Chen, Han Zhang

**Affiliations:** 1 Department of Pediatrics, Shengjing Hospital of China Medical University, Shenyang, China; 2 Department of Pediatrics, Boai Hospital of Zhongshan, Zhongshan, China

**Keywords:** bacterial infection, bronchoscopy, lower respiratory tract infection, mucus plug, *Mycoplasma pneumoniae*, neutrophil-to-macrophage ratio, pathogen burden

## Abstract

**Objective:**

Lower respiratory tract infections (LRTI) remain a leading cause of pediatric hospitalization, yet distinguishing infection from non-infectious airway conditions and identifying the causative pathogen remains a clinical challenge. While bronchoscopy with bronchoalveolar lavage provides direct lower airway sampling, the clinical value of routine bronchoalveolar lavage fluid (BALF) cellular analysis beyond its conventional diagnostic use remains poorly defined.

**Methods:**

This retrospective study included hospitalized children with LRTI who underwent bronchoscopy with BALF cellular analysis and targeted next-generation sequencing, as well as children with foreign-body (FB) aspiration as a noninfectious reference group. First, BALF cellular profiles were compared across three pathogen-defined LRTI subgroups: typical bacterial, viral, and *Mycoplasma pneumoniae* (MP). Second, multivariable linear regression analyses were performed to identify clinical, laboratory, radiologic, and bronchoscopic correlates of BALF cellular composition. Finally, secondary logistic regression analyses were performed to assess the potential clinical utility of BALF cellular composition for identifying LRTI using FB as a reference group and for discriminating typical bacterial LRTI from nontypical bacterial LRTI.

**Results:**

Among 488 children with LRTI, BALF cellular composition differed across pathogen-defined groups, with MP infection showing the most pronounced neutrophil-skewed profile. Higher pathogen burden was independently associated with a more neutrophil-skewed BALF profile, whereas mucus plugs were positively associated with ln(Neu/Mph) in pooled interaction models but showed heterogeneous directions in pathogen-specific models. Exploratory analyses suggested limited species-level variation within the bacterial and viral categories. In secondary analyses, BALF cellular composition showed good discrimination between LRTI and noninfectious group, but provided no significant incremental discrimination for typical bacterial versus nontypical bacterial LRTI when added to clinical variables.

**Conclusion:**

BALF cellular composition showed distinct pathogen-associated patterns in pediatric LRTIs, with MP infection exhibiting the most pronounced neutrophil-skewed profile. Higher pathogen burden was associated with a more neutrophil-skewed profile, while mucus-plug associations were sensitive to model specification. BALF cellular composition discriminated LRTI from noninfectious airway conditions but added no significant discrimination for typical bacterial versus nontypical bacterial LRTI beyond clinical variables.

## 1 Introduction

Lower respiratory tract infections (LRTIs) remain a leading cause of pediatric hospitalization and antimicrobial exposure worldwide, with substantial acute morbidity and, in some children, long-term respiratory sequelae (Sitthikarnkha et al., 2022; Bassat et al., 2023). Etiologic diagnosis is challenging because clinical and radiographic features overlap across bacterial, viral, and atypical pathogens (Meyer Sauteur, 2024). Bronchoscopy with bronchoalveolar lavage enables direct sampling of the lower airways, allowing concurrent microbiologic testing and assessment of local inflammatory responses that are often not captured by upper-airway specimens (Zou et al., 2024; Lin et al., 2025).

Bronchoalveolar lavage fluid (BALF) cellular composition reflects lower-airway immune activity. Neutrophil predominance is typically associated with bacterial infection, whereas viral and atypical pathogens may be associated with lymphocyte-enriched patterns, and macrophage proportions may reflect phagocytic and resolution processes (Davidson et al., 2020; King et al., 2021; Zhu et al., 2025). However, data describing BALF cellular composition in pediatric LRTIs remain limited, and pathogen-specific cellular profiles have not been well established. A key barrier has been imperfect microbiologic classification, as conventional testing in children often fails to identify causative pathogens because of low culture yield, prior antibiotic exposure, and polymicrobial colonization.

Targeted next-generation sequencing (tNGS) has emerged as a complementary approach for pathogen detection in respiratory infections, enabling culture-independent, species-level detection of a broad range of bacterial, viral, and other microbial targets, together with semiquantitative estimates of pathogen load that may facilitate pathogen-based stratification (Chen et al., 2024). However, tNGS detection should not be equated automatically with etiologic causality, as the detection of microbial nucleic acids may reflect active infection, colonization, residual nucleic acids, or contamination. Therefore, tNGS results generally require interpretation in conjunction with the clinical presentation, conventional microbiological and laboratory findings, imaging, and treatment response rather than reliance on sequencing results alone. Despite the increasing use of tNGS in respiratory infections, pediatric studies integrating tNGS-based pathogen characterization with BALF cellular composition remain scarce, and the determinants of BALF cellular patterns and their potential diagnostic utility remain unclear.

To address these gaps, we conducted a tNGS-anchored retrospective cohort study with three complementary aims. First, using tNGS to define pathogen categories, we characterized pathogen-specific BALF cellular signatures; we also compared profiles between infectious and non-infectious conditions. Second, we aimed to identify clinical, laboratory, radiographic, and bronchoscopic factors independently associated with BALF cell-type percentages. Third, we evaluated the diagnostic performance of BALF cellular composition for two clinically relevant tasks: distinguishing infection from non-infection, and differentiating bacterial from nonbacterial LRTIs.

## 2 Materials and methods

### 2.1 Participant enrollment

This retrospective study was conducted at Pediatric Department, Shengjing Hospital of China Medical University, from June 2022 to May 2025. The study protocol was approved by the institutional ethics committee.

We initially screened pediatric inpatients aged 1 month to 18 years who were admitted with a diagnosis of LRTI. The diagnosis was established through a comprehensive evaluation independently performed by two senior respiratory physicians, integrating the following: (1) clinical signs and symptoms; (2) chest imaging findings (CT or radiography); (3) laboratory indices including complete blood count, C-reactive protein, procalcitonin, and conventional pathogen testing; and (4) baseline immune status. Final diagnoses were reassessed and confirmed based on clinical response to treatment. Importantly, BALF differential cell results were not used as part of the clinical diagnostic adjudication. Exclusion criteria included immunodeficiency, use of immunosuppressive therapy, congenital heart disease, neurological or metabolic disorders, hematologic diseases, or malignancies.

As this was a retrospective study, bronchoscopy was performed during routine clinical care based on the treating physician’s clinical judgment rather than according to prespecified study criteria. In this cohort, the main clinical circumstances prompting bronchoscopy included diagnostic uncertainty, unclear microbiological etiology, severe or complicated LRTI, suspected airway obstruction, and significant mucus plugging. We initially screened 15747 hospitalized children with LRTI. From this cohort, we further identified 701 patients who underwent bronchoscopy with both BALF cellular analysis and tNGS results available (**Figure 1**). These children represented a clinically selected subset of the overall LRTI population.

**Figure 1:**
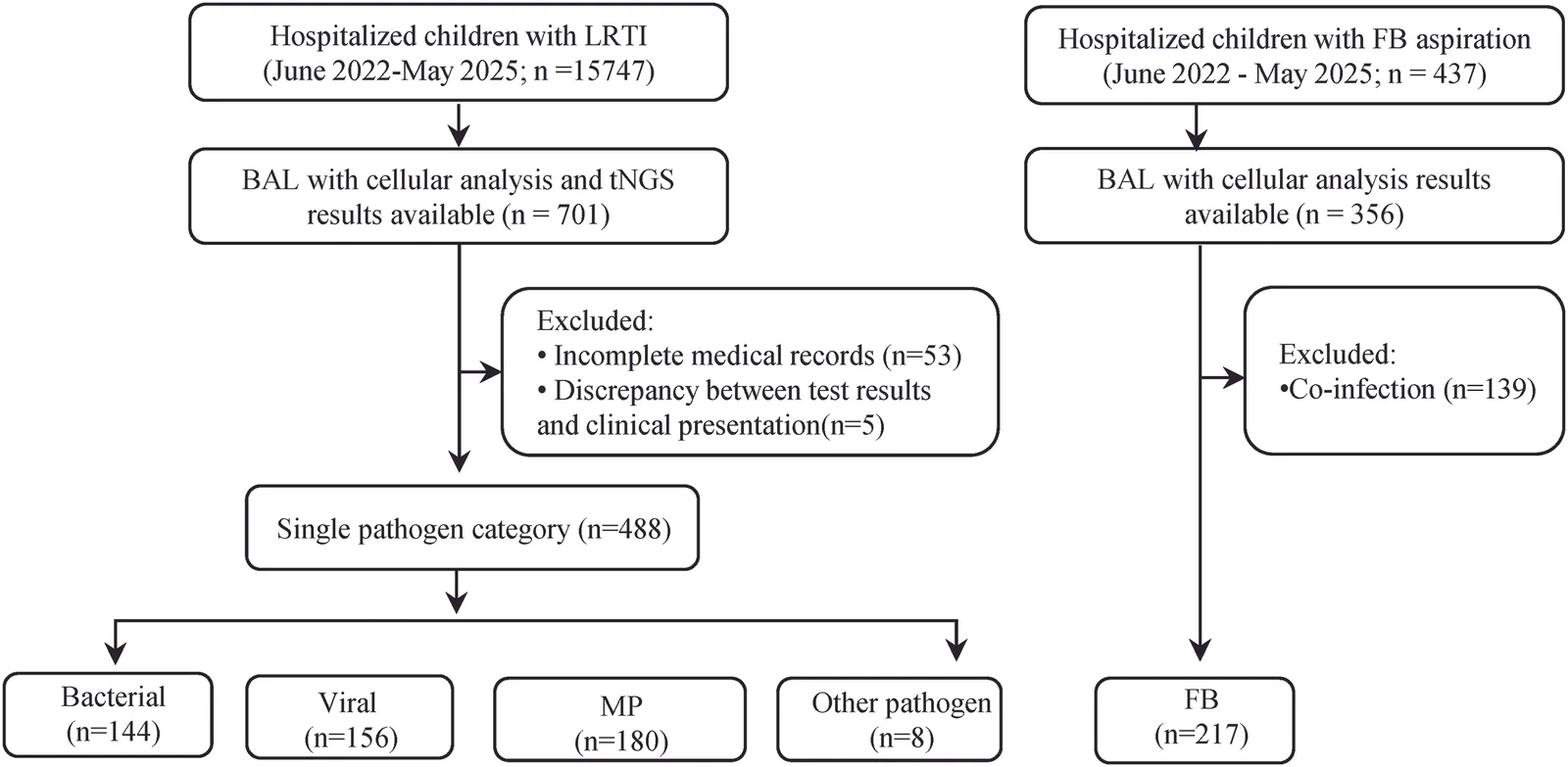
Patient selection flowchart. BAL, bronchoalveolar lavage; FB, foreign body; LRTI, lower respiratory tract infection; MP, Mycoplasma pneumoniae; tNGS, targetednext-generation sequencing.

To evaluate the differences in BALF cellular profiles between infectious and non-infectious airway conditions, we concurrently enrolled a non-infection control group comprising children with foreign body (FB) aspiration during the same study period. Inclusion criteria were: (1) clinical suspicion of FB aspiration, subsequently confirmed by bronchoscopic visualization and successful retrieval of the aspirated foreign body; (2) availability of BALF differential cell counts; and (3) negative microbiological testing with no clinical evidence of concomitant LRTI. A total of 217 patients were ultimately included in the FB group (**Figure 1**).

### 2.2 Clinical data collection

For children in the LRTI group, we collected data on age, sex, time from symptom onset to bronchoscopy, history of atopic disease, and oxygen therapy requirement. Laboratory parameters measured within 7 days before bronchoscopy were retrieved, including white blood cell (WBC) count, C-reactive protein (CRP), and lactate dehydrogenase (LDH). Results of conventional pathogen testing were also collected, comprising: (1) nasopharyngeal swabs for common respiratory virus nucleic acid detection; (2) BALF cultures and smears for conventional bacterial, mycobacterial, and fungal detection; and (3) serum samples for Mycoplasma pneumoniae (MP) and Chlamydia pneumoniae antibody testing. Chest CT findings during the same interval were reviewed and recorded, including consolidation, pleural effusion, atelectasis, and parenchymal necrosis. Radiological findings were assessed by experienced radiologists as part of routine clinical care and documented in the formal radiology reports. Consolidation was defined as increased pulmonary parenchymal opacity with obscuration of the underlying bronchial and vascular structures, with or without air bronchograms. Atelectasis was defined as partial or complete collapse of the lung or a pulmonary lobe, typically accompanied by volume loss. Parenchymal necrosis was defined radiologically as areas of decreased or absent parenchymal enhancement or low attenuation within an area of consolidation, with or without cavitation, consistent with necrotizing parenchymal change. Bronchoscopic findings were likewise documented, encompassing mucus plugs, bronchial occlusion, and bronchiectasis. For children in the FB group, we collected age, sex, atopic status, and oxygen therapy data.

### 2.3 Bronchoscopy and BALF cellular analysis

Bronchoscopy and BAL were not routinely performed in all children with LRTI. Flexible bronchoscopy with bronchoalveolar lavage (BAL) was performed under sedation and topical anesthesia when clinically indicated. After entering the bronchoscopy suite, children received sedation with midazolam. As the bronchoscope was advanced, topical anesthesia was achieved by spraying 1% or 2% lidocaine onto the larynx, the area immediately above the vocal cords, and the carina.

The lavage site was selected based on available chest imaging findings. When a focal abnormality was identified on chest CT, BAL was targeted to the corresponding most severely affected pulmonary segment or lobe. In patients without a clearly localized radiographic abnormality, BAL was performed in the right middle lobe as a standardized non-targeted lavage site. After the bronchoscope was wedged into the target bronchus, sterile normal saline prewarmed to 37 °C was instilled through the working channel in aliquots to a total volume of 1–2 mL/kg. The instilled fluid was then gently aspirated, and at least 30% of the instilled volume was required to be recovered.

A portion of the recovered BALF was immediately submitted for routine clinical testing, including differential cell counts and pathogen testing. BALF differential cell counts were determined in the hospital laboratory as part of routine clinical practice using multiparameter flow cytometry, with standardized gating to identify segmented and band neutrophils, lymphocytes, macrophages, eosinophils, and epithelial cells. Results were reported as percentages.

### 2.4 BALF tNGS processing

An additional 3 mL of BALF was submitted for tNGS analysis, which was performed in a certified laboratory (Hebei Pathogeno Sheng Kang Medical Technology Co., Ltd., China). Library preparation used the Pathogeno One 400+ Library Construction Kit (SJ0009), a multiplex PCR–based targeted panel covering >400 common bacterial pathogens, selected respiratory viruses, and atypical organisms. Preanalytic handling followed a standardized protocol: BALF from the most affected segment or lobe was collected, immediately refrigerated, and transported to the laboratory at 4 °C. Nucleic acids were extracted using the Microbe Magnetic Patho-DNA/RNA Kit (CJ0004), and libraries were prepared according to the manufacturer’s instructions. Sequencing was performed on the Salus Pro platform with run-level quality control thresholds of ≥0.5 million reads per sample, Q30 ≥80%, and single-end 60-bp reads. The assay reported target-specific semiquantitative results as calibrated copies per milliliter (copies/mL) based on internal standards; the analytical limit of detection was 50 copies/mL, and the reportable range was ≥10 reads per million. Each batch included negative and positive controls. Bioinformatic processing included human-read depletion, duplicate removal, and alignment to reference databases (background-filtering database, NCBI RefSeq, and GenBank) using predefined calling thresholds.

### 2.5 Pathogen identification and classification

Pathogens detected by tNGS were considered true-positive findings only when they were concordant with relevant laboratory evidence, treatment response, and the final clinical diagnosis. All etiological results were independently evaluated by two senior respiratory physicians, with a third expert involved in cases of disagreement. Otherwise, they were classified as false positives and excluded from further analysis. The BALF differential cell results were not considered in the pathogen adjudication process.

Based on the pathogen detection results, patients were initially categorized into four etiological categories: typical bacterial, viral, fungal, and atypical pathogens (MP and Chlamydia pneumoniae). The term “typical bacterial” was used to refer to conventional bacterial respiratory pathogens, excluding MP and Chlamydia pneumoniae. MP was subsequently analyzed as a separate subgroup because it represented a substantial proportion of the cohort and showed a distinct clinical and BALF cellular profile compared with the typical bacterial and viral groups. Accordingly, the primary pathogen-defined subgroup analyses focused on three groups: typical bacterial, viral, and MP. Chlamydia pneumoniae and fungal infections were not analyzed as separate subgroups because of their small sample sizes. To enable a clear comparative analysis of distinct etiological profiles, cases with evidence of co-infection involving more than one category were excluded, leaving only those with a single confirmed pathogen category for subsequent subgroup analyses.

Among the LRTI cohort, a total of 488 patients were classified as having pathogens from a single etiological category, including 144 bacterial, 156 viral, 180 MP, 6 fungal cases and 2 Chlamydia pneumoniae (**Figure 1**). Given that bacterial, viral, and MP infections accounted for the vast majority of cases, subsequent analyses focused on these three subgroups.

### 2.6 Statistical analysis

Continuous variables are presented as median (interquartile range), and categorical variables as number (%). For comparisons between two groups, Student’s t test or Mann–Whitney U test was used as appropriate according to data distribution. For comparisons involving three or more groups, the Kruskal–Wallis test was used, followed by Dunn’s test for pairwise comparisons with multiplicity-adjusted P values. Categorical variables were compared using the χ² test or Fisher’s exact test, as appropriate.

For clarity, analyses were classified according to their analytical roles. The primary analyses focused on differences in BALF cellular composition across the three major pathogen-defined LRTI categories (typical bacterial, viral, and MP) and on key clinical and bronchoscopic correlates of the BALF neutrophil-to-macrophage (Neu/Mph) balance, with particular attention to mucus plug formation. Secondary analyses evaluated the ability of BALF cellular features to distinguish LRTI from non-infectious foreign-body aspiration and to provide incremental discrimination between typical bacterial and non-typical bacterial LRTI. Analyses at the individual-pathogen level and pathogen-specific subgroup regression analyses were considered exploratory and hypothesis-generating.

For compositional analyses, the BALF differential count was initially represented by six components: segmented neutrophils, band neutrophils, lymphocytes, macrophages, eosinophils, and epithelial cells. Zero values were handled using a row-wise multiplicative replacement procedure with a replacement value of 0.5, followed by proportional rescaling of the nonzero components to preserve the 100% closure constraint. For the primary analysis, segmented and band neutrophils were combined as total neutrophils (Neu), resulting in a five-part composition. The primary BALF coordinate was defined as the natural logarithm of the Neu/Mph ratio [ln(Neu/Mph)]. In secondary and sensitivity analyses, the original six-part composition was retained, and a complete five-coordinate additive log-ratio (ALR) representation was constructed using macrophages as the reference component. The five coordinates were ln(SegN/Mph), ln(BandN/Mph), ln(Lym/Mph), ln(Eos/Mph), and ln(Epi/Mph). An additional sensitivity analysis used the six original BALF cell fractions as raw compositional variables.

Logistic regression models were used to assess clinical discrimination, with model performance evaluated using receiver operating characteristic curves and area under the curve (AUC). Internal validation was performed using bootstrap resampling where indicated. All tests were two-sided, and P < 0.05 was considered statistically significant for confirmatory comparisons; exploratory analyses were interpreted cautiously with consideration of multiple testing and subgroup sample size.

## 3 Results

### 3.1 Clinical characteristics

Baseline demographic, clinical, laboratory, imaging, and bronchoscopic characteristics of the LRTI group, FB group and pathogen-defined subgroups are summarized in **Table 1**. No serious adverse events related to bronchoscopy or BALF collection were documented in the study cohort. Significant between-group differences were observed across the pathogen-defined LRTI subgroups with respect to age, symptom duration, history of allergic disease, inflammatory markers (CRP and LDH), chest CT findings (consolidation and pleural effusion), and bronchoscopic findings (mucus plugs and bronchiectasis) (**Table 1**).

**Table 1:** Patient characteristics.

Variable	LRTI (n=488)	FB (n=217)	Bacterial (n=144)	Viral (n=156)	MP (n=180)	*p*
Age, y	5 (3-8)	2 (1-2)	4 (2-7) ^b^	4 (2-7) ^c^	7 (5-9)	**<**.**001**
Male	266 (54.5)	132 (60.8)	78 (54.2)	96 (61.5)	87 (48.3)	0.053
Clinical presentation						
Symptom duration^†^, d	21 (12-48)	…^d^	41 (16-100)^ab^	28 (15-60)^c^	13 (11-18)	**<**.**001**
Allergy	228 (46.7)	78 (35.9)	80 (55.6)^b^	82 (52.6) ^c^	62 (34.4)	**<**.**001**
Oxygen therapy	78 (16.0)	10 (4.6)	17 (11.8)	27 (17.3)	32 (17.8)	0.284
Laboratory test						
WBC count, 10^9^/L	8.7 (6.8-11.5)	9.5 (8.2-11.3)	8.6 (7.0-11.4)	8.4 (6.4-11.3)	9.0 (6.9-11.9)	0.441
CRP, mg/L	5.0 (4.9-16.7)	3.3 (1.4-5.0)	5.0 (1.9-9.0) ^b^	5.0 (3.5-9.8) ^c^	11.7 (5.0-27.2)	**<**.**001**
LDH, U/L	276 (236-357)	…^d^	257 (222-300) ^b^	266 (223-310) ^c^	349 (273-467)	**<**.**001**
CT features						
Consolidation	281 (57.6)	…^d^	56 (38.9) ^b^	68 (43.6) ^c^	157 (87.2)	**<**.**001**
Atelectasis	55 (11.3)	…^d^	14 (9.7)	20 (12.8)	21 (11.7)	0.697
Pleural effusion	81 (16.6)	…^d^	4 (2.8) ^a b^	17 (10.9) ^c^	60 (33.3)	**<**.**001**
Necrotic lesions	11 (2.3)	…^d^	6 (4.2)	1 (0.6)	4 (2.2)	0.125
Bronchoscopic findings						
Mucus plugs	230 (47.1)	…^d^	47 (32.6) ^b^	52 (33.3) ^c^	131 (72.8)	**<**.**001**
Bronchial occlusion	20 (4.1)	…^d^	9 (6.3)	7 (4.5)	3 (1.7)	0.101
Bronchiectasis	29 (5.9)	…^d^	18 (12.5) ^a b^	9 (5.8) ^c^	1 (0.6)	**<**.**001**

†Symptom duration was defined as the interval from the onset of the first symptoms to bronchoscopy and BALF collection. Values are median (interquartile range), No. (%). LRTI, lower respiratory tract infection; FB, foreign body; WBC, white blood cell; CRP, C-reactive protein; LDH, lactate dehydrogenase. *P* values represent overall comparisons across the three pathogen-defined LRTI subgroups.

a Bacterial vs Viral, *p* < 0.05.

b Bacterial vs MP, *p* < 0.05.

c Viral vs MP, *p* < 0.05.

d Data not collected.

Bold values indicate statistically significant differences (P < 0.05).

In pairwise comparisons, children in the bacterial subgroup had longer symptom duration and higher frequencies of bronchiectasis than those in the viral subgroup. Children in the MP subgroup were older than those in the other two subgroups and exhibited a distinct clinical profile, characterized by shorter symptom duration, fewer allergic comorbidities, higher CRP and LDH levels, more frequent pulmonary consolidation and pleural effusion, increased mucus plug formation, and a lower prevalence of bronchiectasis (**Table 1**).

### 3.2 BALF cellular composition across major pathogen categories

Distinct BALF cellular patterns were observed across the three major pathogen-defined LRTI subgroups. Notably, segmented neutrophils predominated in the MP subgroup, whereas macrophages remained the dominant cell population in the bacterial and viral subgroups. (**Table 2**; **Figures 2A–C**). Overall, the MP group exhibited substantially higher neutrophil percentages and lower macrophage percentages than the other two subgroups.

**Table 2:** BALF cellular composition in the overall LRTI, FB, and pathogen-defined LRTI subgroups.

Variable	LRTI (n=488)	FB (n=217)	Bacterial (n=144)	Viral (n=156)	MP (n=180)	*p*
Segmented neutrophils, %	28 (10-52)^a^	5 (2-10)	22 (8-48)	19 (7-36)	47 (28-63)	**<**.**001**
Band neutrophils, %	3 (1-7) ^a^	0 (0-2)	2 (1-5)	2 (0-5)	6 (3-11)	**<**.**001**
Lymphocytes, %	8 (4-14) ^a^	5 (3-9)	6 (3-11)	8 (4-16)	9 (5-16)	**<**.**001**
Macrophages, %	48 (24-69) ^a^	80 (72-86)	57 (35-74)	61 (44-72)	27 (15-46)	**<**.**001**
Eosinophils, %	0 (0)	0 (0)	0 (0)	0 (0)	0 (0)	0.890
Epithelial cells, %	2 (0-6) ^a^	5 (2-12)	3 (0-8)	3 (0-8)	0 (0-3)	**<**.**001**

Values are median (interquartile range). LRTI, lower respiratory tract infection; FB, foreign body; *P* values represent overall comparisons across the three pathogen-defined LRTI subgroups.

a LRTI vs FB, *p* < 0.05.

Bold values indicate statistically significant differences (P < 0.05).

**Figure 2:**
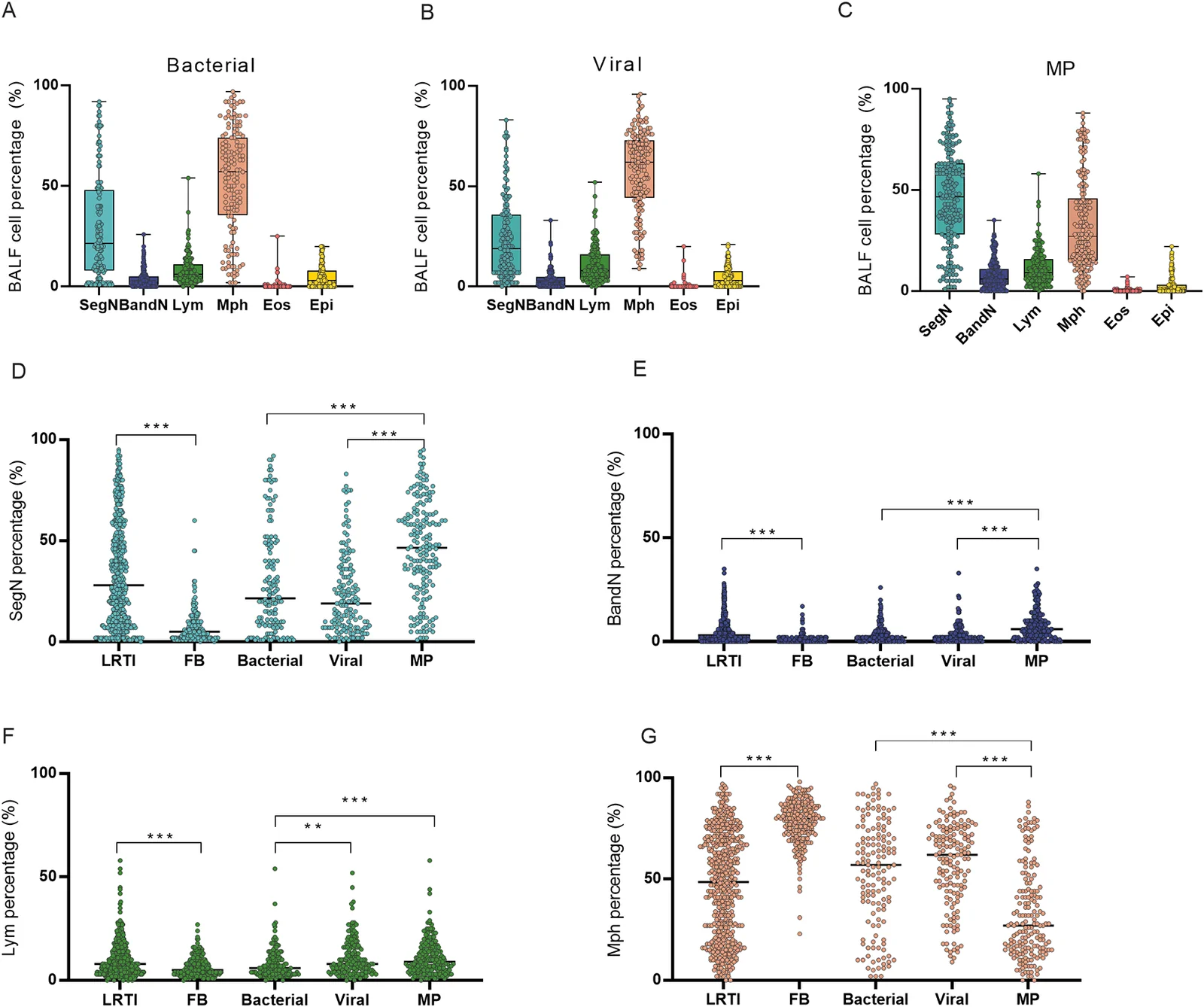
BALF cellular composition in the overall LRTI, FB, and pathogen-defined LRTI subgroups. **(A–C)** Distribution of BALF cell-type percentages in different groups. **(D–G)** Pairwise comparisons of BALF cell percentages across groups. ** indicates P <.01; *** indicates P <.001. BALF, bronchoalveolar lavage fluid; BandN, band neutrophils; Eos, eosinophils; Epi, epithelial cells; FB, foreign body; LRTI, lower respiratory tract infection; Lym, lymphocytes; MP, M pneumoniae; Mph, macrophages; SegN, segmented neutrophils.

Pairwise comparisons further demonstrated that both segmented and band neutrophils were significantly higher in the MP group than in the typical bacterial and viral groups, with no difference between the latter two. Lymphocyte percentages were lowest in the typical bacterial group, whereas macrophage percentages were significantly higher in the typical bacterial and viral groups than in the MP group (**Figures 2D–G**). Together, these findings indicate that BALF cellular composition in pediatric LRTIs differs systematically by pathogen class, with neutrophils and macrophages forming the principal axis of pathogen-associated airway inflammation.

### 3.3 Clinical and bronchoscopic correlates of the BALF Neu/Mph

To identify clinical correlates of BALF cellular profiles, we fitted multivariable linear regression models with BALF neutrophil percentage, macrophage percentage, and the ln(Neu/Mph) as outcomes (neutrophils defined as segmented plus band neutrophils). Across models, pulmonary consolidation, bronchoscopic mucus plugs, bronchial occlusion, and higher pathogen load were consistently associated with a neutrophil-dominant BALF profile, reflected by higher neutrophil percentages, lower macrophage percentages, and an increased ln(Neu/Mph) (**Figures 3A–C**). In contrast, higher peripheral WBC counts were inversely associated with BALF neutrophil percentage and the Neu/Mph ratio, indicating discordance between systemic leukocytosis and local airway inflammation. Collectively, these findings indicate that radiologic and bronchoscopic markers of airway involvement, together with pathogen burden, are key determinants of BALF cellular composition in pediatric LRTIs.

**Figure 3:**
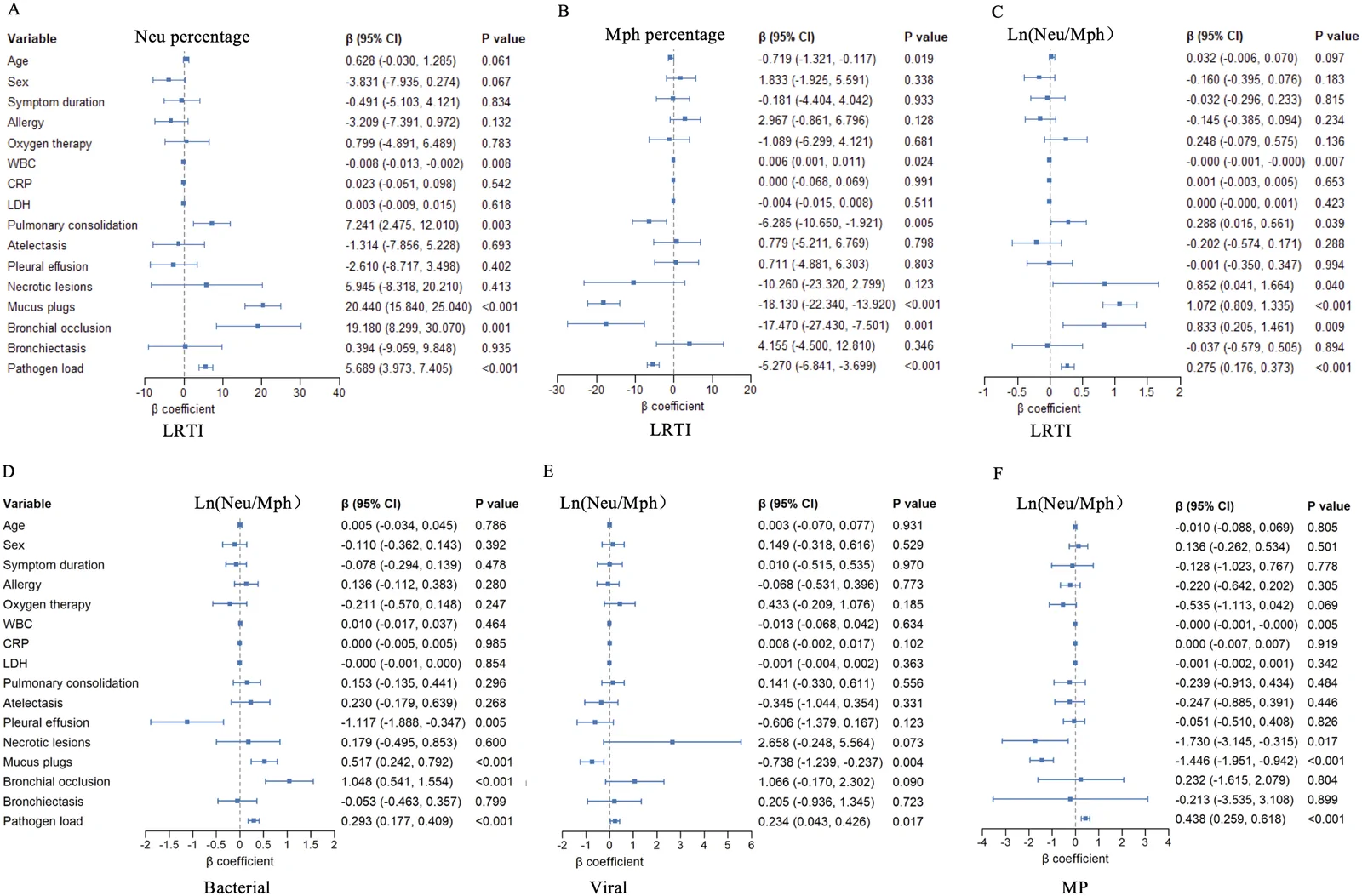
Multivariable regression models of BALF cellular composition in the overall LRTI cohort and pathogen-defined subgroups. **(A–C)** Forest plots show multivariable linear regression coefficients (β) and 95% CIs for factors associated with BALF neutrophil percentage, macrophage percentage, and ln(Neu/Mph), respectively, in the overall LRTI cohort. **(D–F)** Forest plots show multivariable linear regression coefficients (β) and 95% CIs from models using ln(Neu/Mph) as the dependent variable in the typical bacterial, viral, and MP groups, respectively. BALF, bronchoalveolar lavage fluid; CI, confidence interval; LRTI, lower respiratory tract infection; Mph, macrophages; Neu, neutrophils; MP, Mycoplasmapneumoniae.

### 3.4 Pathogen burden and mucus plugs are associated with neutrophil-skewed BALF profiles

In the overall LRTI cohort, multivariable analyses showed that higher pathogen load and the presence of mucus plugs were independently associated with a neutrophil-skewed BALF cellular profile, characterized by a higher Neu/Mph ratio (**Figures 3A–C**). To further examine whether these associations were consistent across pathogen-defined groups, we performed exploratory subgroup analyses using ln(Neu/Mph) as an integrated outcome, given the reciprocal patterns of neutrophil and macrophage percentages. Separate multivariable models were fitted within the typical bacterial, viral, and MP groups.

When examined within pathogen-defined subgroups, higher pathogen load was consistently associated with a higher ln(Neu/Mph) (**Figures 3D–F**). Mucus-plug estimates, however, varied in direction across the separately fitted pathogen-specific models (**Figures 3D–F**), indicating sensitivity of the adjusted association to subgroup-specific model estimation. To further assess the robustness of this finding, we fitted pooled models including a mucus plug × pathogen-category interaction, thereby allowing the mucus-plug association to vary across pathogen categories while estimating common effects for the other covariates. In both minimally and fully adjusted pooled models, mucus plugs showed positive associations with ln(Neu/Mph) in the typical bacterial, viral, and MP groups. In the fully adjusted model, the corresponding exp(β) values were 3.54 (95% CI, 1.94–6.45), 1.93 (95% CI, 1.24–3.01), and 4.67 (95% CI, 2.74–7.95), respectively (**Supplementary Figure S1**). These findings provide supportive evidence for a positive association between mucus plugs and a neutrophil-skewed BALF profile, while indicating that the magnitude and direction of subgroup-specific adjusted estimates may depend on model specification.

We next performed descriptive analyses to visualize these relationships. Across the typical bacterial-, viral-, and MP groups, higher pathogen load was associated with higher BALF neutrophil percentages and lower macrophage percentages (**Figures 4A, B, D, E, G, H**). Similarly, children with mucus plugs showed higher neutrophil and lower macrophage percentages than those without mucus plugs across all three groups, whereas lymphocyte percentages showed no clear differences according to mucus plug status (**Figures 4C, F, I**). These descriptive findings were consistent with the pooled interaction analyses and further supported an association between pathogen burden and mucus plug formation with a neutrophil-skewed BALF cellular profile.

**Figure 4:**
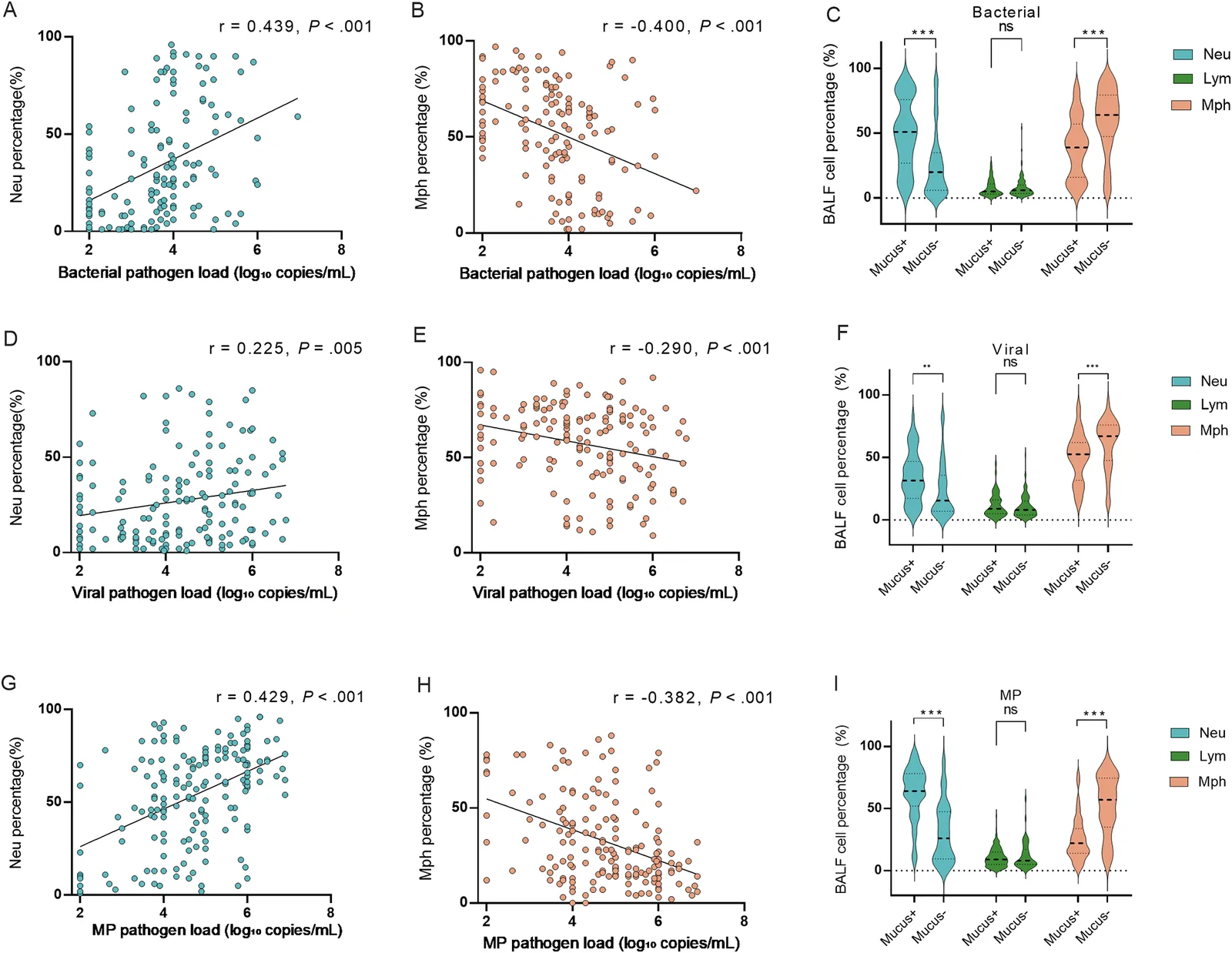
Pathogen load and mucus plugs are associated with a neutrophil-skewed BALF profile. Scatterplots depict associations between pathogen load and BALF neutrophil percentage **(A, D, G)** and macrophage percentage **(B, E, H)**. Violin plots compare BALF cellular composition by mucus plug status within each subgroup **(C, F, I)**. Neutrophils represent the sum of segmented neutrophils and band neutrophils. Neu, neutrophils; Lym, lymphocytes; Mph, macrophages.

### 3.5 Exploratory analysis of pathogen-specific BALF cellular composition

As an exploratory analysis, we examined whether BALF cellular composition differed among individual pathogens within the same etiologic category. Analyses were performed separately within the typical bacterial and viral groups.

Within the typical bacterial group, the five most frequently detected pathogens were Haemophilus influenzae (n = 44), Streptococcus pneumoniae (n = 39), Staphylococcus aureus (n = 12), Moraxella catarrhalis (n = 12), and Pseudomonas aeruginosa (n = 9), with other bacterial pathogens detected less frequently. Within the viral group, the five most frequently detected pathogens were respiratory syncytial virus (RSV; n = 32), rhinovirus (n = 32), parainfluenza virus type 3 (n = 19), human adenovirus (n = 17), and human bocavirus (n = 14), with other viral pathogens detected less frequently.

Among the five most frequently detected bacterial pathogens, no significant differences were observed in segmented neutrophil, band neutrophil, lymphocyte, or macrophage percentages (**Table 3**). Among the five most frequently detected viral pathogens, overall differences were observed in segmented neutrophil and macrophage percentages, whereas band neutrophil and lymphocyte percentages did not differ significantly. Pairwise comparisons showed that the RSV group had higher segmented neutrophil percentages and lower macrophage percentages than the human bocavirus group; no other pairwise differences were detected (**Table 3**).

**Table 3:** Within-category pathogen comparisons of BALF cellular composition.

Panel A. Bacterial subgroup (Top 5 pathogens)						
Variable	*Haemophilus influenzae* (n=44)	*Streptococcus pneumoniae* (n=39)	*Staphylococcus aureus* (n=12)	*Moraxella catarrhalis* (n=12)	*Pseudomonas aeruginosa* (n=9)	*p* ^a^
Segmented neutrophils, %	29 (11-58)	20 (7-39)	21 (6-48)	21 (5-50)	10 (5-56)	0.776
Band neutrophils, %	4 (2-7)	2 (0-5)	2 (1-4)	2 (1-4)	2 (0-5)	0.203
Lymphocytes, %	6 (3-12)	7 (4-12)	5 (3-10)	5 (3-12)	5 (4-15)	0.734
Macrophages, %	51 (19-70)	63 (39-77)	61 (30-76)	55 (43-78)	56 (30-78)	0.689
Panel B. Viral subgroup (Top 5 viruses)						
Variable	Respiratory syncytial virus (n=32)	Rhinovirus (n=32)	Parainfluenza virus (n=19)	Adenovirus (n=17)	Human bocavirus (n=14)	*p* ^b^
Segmented neutrophils, %	31 (16-43)	24 (11-43)	14 (7-27)	15 (9-26)	7 (4-17)	**<**.**001**
Band neutrophils, %	3 (1-7)	3 (1-4)	2 (0-4)	2 (1-5)	1 (0-3)	0.267
Lymphocytes, %	8 (3-17)	8 (3-13)	8 (6-13)	11 (5-20)	14 (6-17)	0.461
Macrophages, %	48 (32-63)	52 (38-71)	63 (53-72)	56 (50-74)	69 (60-75)	**0.016**

a *p* values represent overall comparisons across the five bacterial pathogens.

b *p* values represent overall comparisons across the five viral pathogens.

Bold values indicate statistically significant differences (P < 0.05).

Overall, no consistent differences in BALF cellular composition were detected among the most frequently identified individual bacterial pathogens, whereas limited differences were observed among selected viral pathogens. Given the exploratory nature of these analyses and the relatively small sample sizes of several pathogen-specific subgroups, these findings should be interpreted cautiously as hypothesis-generating rather than as evidence against species-level differences.

### 3.6 Secondary analysis: BALF cell-based model for identifying LRTI

To explore the potential clinical utility of BALF cellular composition in identifying LRTI when the clinical presentation is atypical or microbiological diagnosis remains unavailable or inconclusive, we performed a secondary prediction analysis using children with noninfectious FB group as a reference group. The purpose of this analysis was not to establish a clinically relevant diagnostic distinction between LRTI and FB, but to assess whether BALF cellular composition provides information that may support recognition of LRTI in diagnostically challenging cases.

Because BALF differential counts are compositional, the ln(Neu/Mph) was used as the primary BALF predictor. The dataset was randomly divided into training (70%) and test (30%) sets. The ln(Neu/Mph)-based model showed good discrimination, with an AUC of 0.865 in the training set and 0.824 in the test set (**Figure 5A**). A nomogram based on ln(Neu/Mph) was constructed to graphically estimate the predicted probability of LRTI according to the observed BALF Neu/Mph ratio (**Figure 5B**).

**Figure 5:**
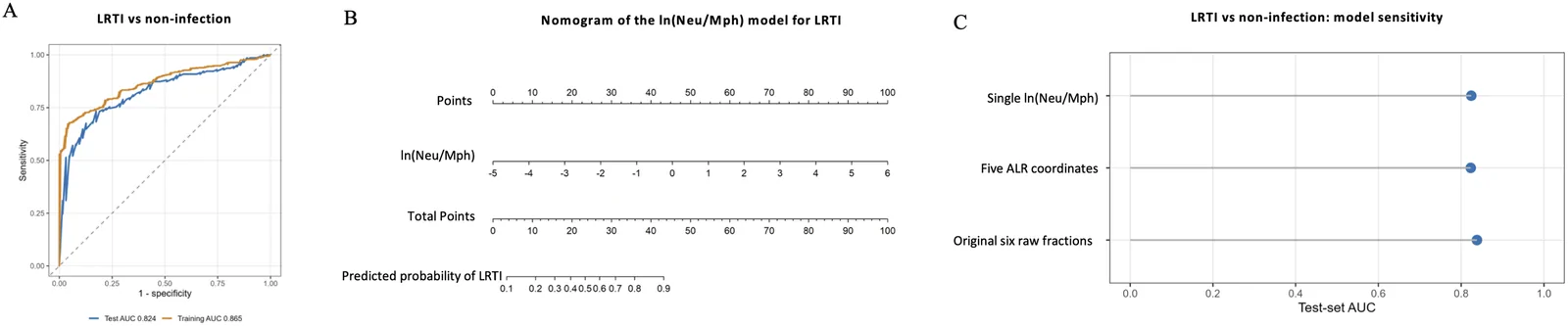
Diagnostic performance and sensitivity analyses of BALF cellular composition for identifying LRTI. **(A)** Receiver operating characteristic (ROC) curves for the BALF cellular composition model based on ln(Neu/Mph) for identifying LRTI, using children with foreign-body aspiration as the noninfectious reference group. **(B)** Nomogram based on ln(Neu/Mph) for estimating the predicted probability of LRTI. **(C)** Sensitivity analysis comparing the test-set discrimination of models using three alternative representations of BALF cellular composition: single ln(Neu/Mph), five additive log-ratio (ALR) coordinates, and the original six BALF cell fractions. BALF, bronchoalveolar lavage fluid; ALR, additive log-ratio; LRTI, lower respiratory tract infection; Neu, neutrophils; Mph, macrophages; ROC, receiver operating characteristic.

To assess the robustness of model performance to alternative representations of the compositional BALF data, we performed sensitivity analyses using five ALR coordinates and the original six raw cell fractions as alternative predictor sets. The corresponding test-set AUCs were 0.823 for the five-ALR-coordinate model and 0.837 for the model using the original six raw fractions, compared with 0.824 for the single ln(Neu/Mph) model (**Figure 5C**). The similar discrimination across these alternative representations supported the robustness of the observed predictive performance to the choice of BALF cellular representation.

These findings suggest that BALF cellular composition contains potentially useful information for identifying LRTI in diagnostically challenging cases. However, this secondary analysis was intended to evaluate potential clinical utility rather than establish a definitive diagnostic tool, and BALF cellular composition should not be considered a substitute for microbiological testing.

### 3.7 Secondary analysis: added value of BALF cellular composition for identifying typical bacterial LRTI

As a secondary analysis, we evaluated whether BALF cellular composition provided additional discriminatory value for identifying typical bacterial LRTI among children with LRTI. Thirteen clinical, laboratory, radiologic, and bronchoscopic variables that differed between the typical bacterial LRTI and non-typical bacterial LRTI groups in univariable analyses were entered into a least absolute shrinkage and selection operator logistic regression model. Using the 1-SE criterion, four predictors—symptom duration, consolidation, pleural effusion, and bronchial occlusion—were retained to construct the clinical Model 1.

Model 1 showed moderate discrimination, with an AUC of 0.750 (95% CI, 0.703–0.797). To assess the incremental value of BALF cellular composition, ln(Neu/Mph) was subsequently added to the clinical model. The resulting Model 2 showed an AUC of 0.750 (95% CI, 0.704–0.797), with no improvement in discrimination compared with Model 1 (**Figure 6A**).

**Figure 6:**
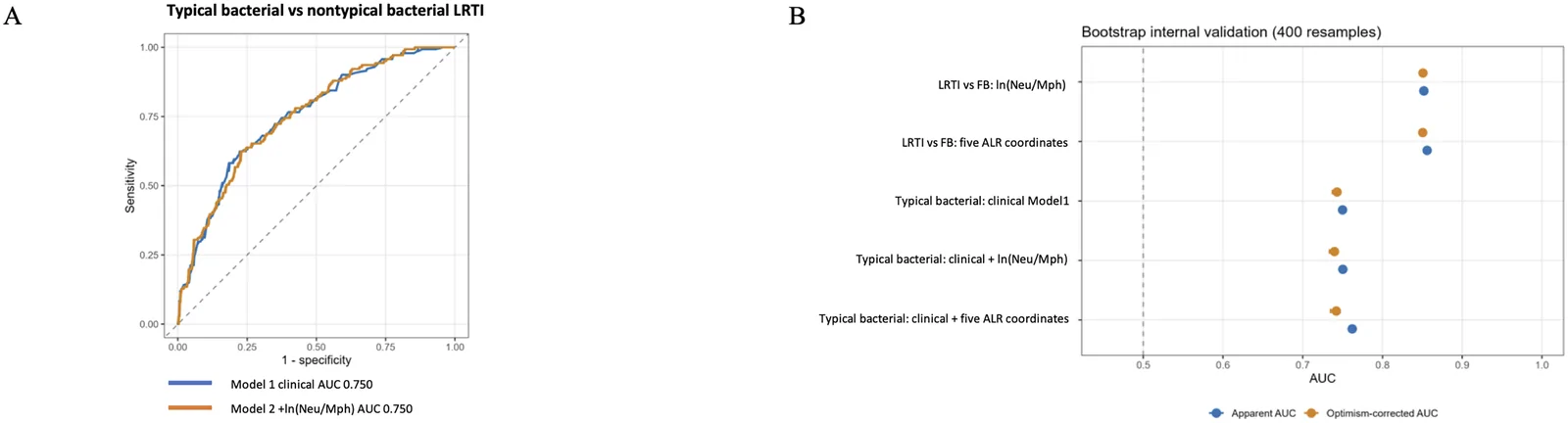
Performance and internal validation of models for identifying typical bacterial LRTI. **(A)** Receiver operating characteristic (ROC) curves comparing the clinical Model 1 and the clinical Model 2 incorporating ln(Neu/Mph) for identifying typical bacterial LRTI versus nontypical bacterial LRTI. The AUCs were 0.750 for both models. **(B)** Bootstrap internal validation comparing the apparent and optimism-corrected AUCs across alternative model specifications. For LRTI versus foreign-body aspiration, models based on ln(Neu/Mph) and five additive log-ratio (ALR) coordinates were evaluated. For typical bacterial versus nontypical bacterial LRTI, the clinical Model 1, clinical Model 2 incorporating ln(Neu/Mph), and a model incorporating five ALR coordinates were evaluated. AUC, area under the receiver operating characteristic curve; ALR, additive log-ratio; BALF, bronchoalveolar lavage fluid; LRTI, lower respiratory tract infection; Neu, neutrophils; Mph, macrophages; ROC, receiver operatingcharacteristic.

To assess the robustness of these findings to alternative representations of the compositional BALF data, we further performed a sensitivity analysis incorporating the complete five-coordinate additive log-ratio (ALR) representation of BALF cellular composition together with the clinical predictors. This model yielded an AUC of 0.762, which was not significantly different from either the clinical Model 1 (DeLong test, P = 0.211) or Model 2 incorporating ln(Neu/Mph) (P = 0.189). Thus, the use of the complete ALR representation did not provide evidence of additional discriminatory information beyond the clinical predictors.

Bootstrap internal validation with 400 resamples yielded optimism-corrected AUCs of 0.743 for the clinical Model 1, 0.740 for Model 2 incorporating ln(Neu/Mph), and 0.742 for the model incorporating the five ALR coordinates (**Figure 6B**). The similar optimism-corrected AUCs across the three models further supported the stability of the finding that incorporating BALF cellular composition did not materially improve discrimination.

Overall, routine clinical, radiologic, and bronchoscopic features accounted for the major discriminatory information for distinguishing typical bacterial LRTI from non-typical bacterial LRTI, whereas the addition of BALF cellular composition did not provide clear incremental discrimination in this cohort.

## 4 Discussion

In healthy adults, BALF is dominated by alveolar macrophages and lymphocytes, with neutrophils typically comprising only a minor fraction (Meyer et al., 2012). Infection represents a major driver of deviation from this baseline pattern, and in adults, infectious pulmonary diseases are consistently associated with higher BALF neutrophil proportions and neutrophil-to-lymphocyte ratios compared with noninfectious conditions (Pan et al., 2022). Whether similar immune shifts characterize pediatric lower-airway disease has been less well defined. In this study, we demonstrate that pediatric LRTIs are likewise associated with a pronounced shift toward neutrophil predominance and relative macrophage depletion compared with noninfectious airway obstruction due to FB aspiration, supporting the relevance of BALF cytology as a marker of infection-associated airway inflammation in children.

Prior adult studies have shown that BALF cellular patterns vary across infectious etiologies, with bacterial pneumonia generally associated with higher neutrophil fractions than viral infections, although organism-specific profiles remain incompletely characterized (Choi et al., 2014). In coronavirus disease 2019 (COVID-19), BALF findings have been heterogeneous, ranging from marked neutrophilia in severe disease to lymphocyte-enriched patterns in other cohorts (Liao et al., 2020; Gelarden et al., 2021). Similar heterogeneity has also been described in other viral infections, including neutrophil-predominant patterns in acute adenovirus infection, an early neutrophilic phase followed by lymphocytosis in influenza, and concurrent increases in neutrophils and lymphocytes in parainfluenza infection (Faverio et al., 2014; Pokharel et al., 2014; Campbell et al., 2017). Together, these data suggest that BALF cellular composition reflects a balance between pathogen burden, host immune activation, and disease stage rather than a single uniform response.

Pediatric data have been more limited and largely confined to a narrow set of pathogens. Studies of MP pneumonia, particularly severe or refractory disease, have consistently described neutrophil-predominant BALF profiles with reduced macrophages (Deng et al., 2023; Zhang et al., 2023; Ma et al., 2024). Reports in other pediatric infections have suggested variability, including predominantly neutrophilic patterns in respiratory syncytial virus infection and relatively lower neutrophil proportions in human bocavirus–associated disease (McNamara et al., 2003; Liu et al., 2024). These observations underscore the need for systematic, pathogen-stratified analyses in pediatric LRTIs.

By integrating tNGS-based pathogen classification with BALF cytology, our study extends prior work by characterizing BALF cellular patterns across broad pathogen-defined groups and exploring their variation among individual pathogens within each group. Macrophages and segmented neutrophils predominated in bacterial and viral infections, whereas segmented neutrophils were most prominent in MP group. In contrast, within-category comparisons showed relatively limited evidence of species-level differences, although the small sample sizes of several pathogen groups preclude firm conclusions regarding organism-specific cellular patterns.

Beyond pathogen classification, we found that BALF cellular composition was closely linked to pathogen burden and bronchoscopic markers of airway injury. Across the overall LRTI cohort, higher pathogen burden and the presence of mucus plugs were independently associated with a more neutrophil-skewed BALF cellular profile, characterized by higher neutrophil and lower macrophage proportions. Other clinical and bronchoscopic features, including consolidation and bronchial occlusion, showed additional associations with BALF cellular composition. This reciprocal pattern is biologically plausible. Increased neutrophil proportions may reflect pathogen-associated innate immune activation and epithelial injury, whereas the relative reduction in macrophage proportions may reflect both the influx of neutrophils and alterations in macrophage homeostatic functions during acute inflammation (Palmer and Kimmey, 2022; Mahida et al., 2021; Malainou et al., 2023). The Neu/Mph ratio therefore provides an integrated index of this cellular shift.

When examined within pathogen-defined subgroups, higher pathogen burden was consistently associated with a more neutrophil-skewed BALF profile. In pooled interaction models, the presence of mucus plugs was positively associated with ln(Neu/Mph) across the typical bacterial, viral, and MP groups, although estimates from separately fitted pathogen-specific models varied in direction. Mucus plugs may represent a morphologic manifestation of neutrophil-driven airway inflammation, potentially involving neutrophil elastase–mediated mucin hypersecretion and stabilization of airway casts by neutrophil extracellular traps (Linssen et al., 2021; Voynow and Shinbashi, 2021; Keir and Chalmers, 2022; Liegeois et al., 2025). Similarly, higher pathogen burden may promote neutrophil recruitment through pathogen-associated molecular patterns and downstream proinflammatory signaling (Nathan, 2006; Sadik et al., 2011). Together, these findings support a potential link between microbial burden, mucus plugging, and local neutrophilic polarization in pediatric LRTIs, while the cross-sectional design does not establish a temporal or causal relationship.

The heterogeneous estimates from the separately fitted pathogen-specific models should be interpreted cautiously. These models estimated all covariate effects independently within each pathogen group, whereas the pooled interaction model allowed the mucus-plug association to differ by pathogen category while assuming common effects of the other covariates across groups. Differences in model parameterization, together with limited subgroup sample sizes and correlations among mucus plugging, airway obstruction, radiographic abnormalities, and pathogen burden, may have contributed to variation in the estimated subgroup-specific associations. Thus, the positive associations observed in the pooled interaction model should be considered supportive rather than definitive evidence of a pathogen-independent relationship between mucus plugging and a neutrophil-skewed BALF profile.

BALF cellular composition may provide supportive information for identifying LRTI in diagnostically challenging cases, but should not be considered a substitute for microbiological testing. BALF cellular analysis is well established as a marker of disease activity and prognosis in interstitial lung diseases (Kono et al., 2021; Lee et al., 2025),although its clinical interpretation requires caution (Grant-Orser et al., 2025).In adults pulmonary infection, a BALF neutrophil percentage ≥ 16% has been proposed to help distinguish infectious from noninfectious etiologies (Li et al., 2024), and combining BALF NGS with BALF neutrophil percentage or the neutrophil-to-lymphocyte ratio improves discrimination (Pan et al., 2022). However, its diagnostic value has been less extensively studied in children. In our secondary analysis, BALF cellular composition showed good discrimination between children with LRTI and those with foreign-body aspiration. Although this comparison was not intended to establish a clinically relevant diagnostic distinction, the findings suggest that BALF cellular composition may provide supportive information in selected diagnostically challenging cases. In contrast, for differentiating typical bacterial from nontypical bacterial LRTIs, routinely available clinical and imaging features captured most of the discriminatory signal, and BALF cytology offered only limited incremental improvement. These findings suggest that BALF cellular analysis is best viewed as complementary, with potential value in selected or diagnostically challenging cases rather than as a standalone etiologic discriminator.

Several limitations should be considered. First, this was a single-center, bronchoscopy-based retrospective cohort in which BAL and tNGS were performed based on clinical indications. Consequently, the study population was likely enriched for children with more severe, complicated, or diagnostically challenging presentations, introducing potential selection bias and limiting generalizability to the broader pediatric LRTI population. In particular, mucus plugging may contribute to airway obstruction and distal atelectatic or consolidative changes, which may themselves increase the likelihood of bronchoscopy; therefore, the observed association between mucus plugging and a neutrophil-skewed BALF profile should not be interpreted as causal. In addition, pathogen load derived from tNGS was semiquantitative, and the pathogen-predominance definition excluded cases without clear dominance, potentially limiting representation of less common or mixed infections.

Second, potential residual confounding and incorporation bias warrant consideration. Antimicrobial and corticosteroid exposure before BALF collection was not systematically recorded and could have influenced BALF cellular composition. Although BALF differential cell results were not used for pathogen adjudication, some clinical and radiologic variables included in subsequent prediction models also contributed to the clinical reference diagnosis; therefore, the predictive models were not fully independent of the reference standard and their discrimination should be interpreted cautiously. Statistical constraints also limited the ability to fully model determinants of BALF lymphocyte variation. Finally, several organism-specific groups were small; thus, the absence of statistically significant species-level differences does not exclude potentially meaningful differences. Future multicenter, prospective studies with standardized bronchoscopy criteria, time-resolved treatment documentation, longitudinal sampling, and independently adjudicated microbiological reference standards are needed to validate these findings and further clarify the role of BALF cytology in etiologic assessment and in distinguishing infection from colonization.

## 5 Conclusions

This study characterizes pathogen-associated BALF cellular patterns in pediatric LRTIs, with MP infection showing a particularly pronounced neutrophil-skewed profile. Higher pathogen burden was independently associated with a more neutrophil-skewed BALF profile, whereas the association between mucus plugs and ln(Neu/Mph) was sensitive to model specification. BALF cellular composition showed good discrimination between LRTI and noninfectious airway conditions in a secondary analysis, but provided only modest additional discrimination for identifying typical bacterial LRTI versus nontypical bacterial LRTI. Overall, these findings indicate that BALF cellular composition may provide complementary information for pathogen-associated characterization and clinical assessment of pediatric LRTIs, while not serving as a substitute for microbiological testing.

## Data Availability

The data analyzed in this study is subject to the following licenses/restrictions: The data were obtained from the electronic medical records and laboratory information system of Shengjing Hospital, China Medical University, and contain potentially identifying patient information. Due to hospital institutional policies and patient confidentiality requirements, these data are not publicly available. Access may be granted upon reasonable request to the corresponding authors, subject to institutional review and approval. Requests to access these datasets should be directed to zhangh7626@163.com.
