# Supplementary material for: BALF cellular composition in pediatric LRTI: from pathogen-specific patterns to clinical correlates
Source: Front Cell Infect Microbiol. 2026 Sep 29;16:1946336. doi: 10.3389/fcimb.2026.1946336 (PMC13657955; doi:10.3389/fcimb.2026.1946336)
Supplement: Supplementary file 1 [file Supplementaryfile1.docx]

Supplementary Material

**Supplementary Figure S1. Pooled multivariable analyses of factors associated with the BALF neutrophil-to-macrophage ratio across pathogen-defined groups.**

(A–C) Forest plots show associations of clinical variables with the BALF Neu/Mph ratio in the typical bacterial, viral, and MP groups, respectively, using minimally adjusted models incorporating a mucus plug × pathogen category interaction term. (D–F) Forest plots show the corresponding associations from fully adjusted models incorporating clinical, laboratory, radiologic, and bronchoscopic variables. Estimates are presented as exponentiated regression coefficients [exp(β)] with 95% CIs and P values, representing the multiplicative change in the adjusted Neu/Mph ratio associated with each variable. BALF = bronchoalveolar lavage fluid; CI = confidence interval; LRTI = lower respiratory tract infection; MP = Mycoplasma pneumoniae; Neu = neutrophils; Mph = macrophages.
